# The electron-phonon interaction at deep Bi_**2**_Te_3_-semiconductor interfaces from Brillouin light scattering

**DOI:** 10.1038/s41598-017-16313-5

**Published:** 2017-11-27

**Authors:** M. Wiesner, A. Trzaskowska, B. Mroz, S. Charpentier, S. Wang, Y. Song, F. Lombardi, P. Lucignano, G. Benedek, D. Campi, M. Bernasconi, F. Guinea, A. Tagliacozzo

**Affiliations:** 1 Faculty of Physics, Adam Mickiewicz University, Umultowska 85, PL61614 Poznan, Poland; 2 The NanoBioMedical Centre, Adam Mickiewicz University, Umultowska 85, PL, 61614 Poznan, Poland; 3 Department of Microtechnology and Nanoscience, Chalmers University of Technology, SE-412 96, Göteborg, Sweden; 4 State Key Laboratory of Functional Materials for Informatics, Shanghai Institute of Microsystem and Information Technology, Chinese Academy of Sciences, 865 Changning Road, Shanghai, 200050 China; 5 CNR-SPIN, Monte S. Angelo-Via Cintia, I-80126 Napoli, Italy; 6 Dipartimento di Fisica, Università di Napoli Federico II, Via Cintia, I-80126 Napoli, Italy; 7 Donostia International Physics Centre (DIPC), Paseo Manuel de Lardizábal 4, 20018 Donostia/San, Sebastian, Spain; 8 Dipartimento di Scienza dei Materiali, Università di Milano-Bicocca, Via Cozzi 53, 20125 Milano, Italy; 9 Imdea Nanociencia, Faraday 9, Cantoblanco, 28049 Madrid, Spain; 10 School of Physics and Astronomy, University of Manchester, Manchester, M13 9PL UK; 11 INFN, Laboratori Nazionali di Frascati, Via E.Fermi, Frascati, Italy

## Abstract

It is shown that the electron-phonon interaction at a conducting interface between a topological insulator thin film and a semiconductor substrate can be directly probed by means of high-resolution Brillouin light scattering (BLS). The observation of Kohn anomalies in the surface phonon dispersion curves of a 50 nm thick Bi_2_Te_3_ film on GaAs, besides demonstrating important electron-phonon coupling effects in the GHz frequency domain, shows that information on deep interface electrons can be obtained by tuning the penetration depth of optically-generated surface phonons so as to selectively probe the interface region, as in a sort of *quantum sonar*.

## Introduction

The electron-phonon (e-ph) interaction at the interface of supported thin films plays an important role in the electronic transport. The same holds for signal processing devices based on high-frequency surface acoustic waves (SAWs) filtering devices and resonators^1^, and for heat transport.

A relevant case is that of heat transmission through the interface between a semiconductor, where phonons are the main heat carriers, and a conducting film, where heat conductivity is mainly electronic. The knowledge of interface e-ph interaction has great relevance in the engineering of low-power nanoelectronics as well as in thermoelectric materials such as Bi_2_Te_3_ and Bi_2_Se_3_ films epitaxially grown on semiconductors^2^. Here e-ph interaction needs to be known and controlled in order to achieve a high thermoelectric performance^3^. Bi_2_Te_3_ has long since been known for having the highest figure of merit among thermoelectric materials^4^ and holds promises for feasible solar-cell applications^5,6^ and thermoelectric nanodevices^7,8^. At the interface between two semiconductors the electronic transport properties largely depend on the space charge produced by the work-function difference and the consequent charge transfer across the interface. In the specific case of the Bi_2_Te_3_/GaAs interface a negative space charge of conduction electrons is formed within the interface quintuple layer (QL) of Bi_2_Te_3_. It is the e-ph interaction of these electrons which is of special interest for the properties and applications mentioned above and constitutes the subject of the present study.

More recently Bi_2_Te_3_ and Bi_2_Se_3_ have come into the limelight also for their topological insulator (TI) properties, characterized by the presence of topologically protected helical states localized at a free surface or at the interface with a topologically trivial material^8–16^. The relevance of topologically protected electronic states in electronic transport properties has been widely emphasized^17–20^ and a tool to explore their e-ph interaction at the TI/semiconductor interface should be very useful.

Recent three-pulse photoacoustic spectroscopic studies by Liao *et al*.^21^ on the damping of single sub-THz phonons by free electrons in silicon underlined the importance of directly measuring the mode-selected e-ph interaction in the GHz domain to gain knowledge on phonon transport above cryogenic temperatures, especially for applications in thermoelectrics and photovoltaics. In this paper we demonstrate that Brillouin light scattering (BLS) from single surface phonons can be used to investigate mode-selected e-ph interactions in the GHz domain at the interface of a TI topological insulator thin film epitaxially grown on a semiconductor substrate. Surface acoustic phonons like Rayleigh waves (RWs) propagating at the surface of a semi-infinite homogeneous elastic medium obey a scaling law which makes their penetration depth proportional to the surface wavelength 2π/*q*, i.e., inversely proportional to their frequency^22^. In the GHz frequency range accessible to BLS, the penetration depth of RWs is typically between 10 to 100 nm. In a BLS experiment on a supported film, the RW frequency can therefore be tuned so that the penetration depths matches the film thickness. At this frequency the phonon strain field and the associated e-ph deformation potential have their maxima just in the space charge region along the interface. The same holds true for the longitudinal acoustic resonance (LR)^23^ and the lowest-order Sezawa waves (SWs), which occur in the film which is deposited on a stiffer substrate^24,25^. Thus BLS spectroscopy is a choice method to measure the dispersion curves in that frequency range and to explore the e-ph interaction at the film-substrate interface by tuning BLS to those phonon frequencies whose displacement gradient at the interface is the largest. The tuning is realized by varying the laser beam scattering geometry while measuring the photon energy loss or gain after the inelastic scattering, as done in current BLS spectroscopy.

The observation of a comparatively large softening of the phonon frequencies around the wavevector fulfilling such tuning conditions allows to measure the e-ph coupling of the interface electronic states with the phonon modes having the given wavevector. The latter can in turn be changed by changing the film thickess, so that the observed anomalies in the dispersion curves may be termed as *structural anomalies*.

The logical structure of this report is the following. The first Section (*BLS data*) presents the BLS measurements of the phonon dispersion curves in the GHz spectral region for epitaxially-grown Bi_2_Te_3_ (111)/GaAs(001) films of two different thicknesses, and the changes induced by an external orthogonal magnetic field. Unlike the smooth dispersion relations predicted by the elastic theory of supported thin films, the experiment shows a pronounced softening in frequency and phase velocity around a specific surface wavevector, which is shown to be that of phonons with the largest strain at the interface. In the second Section (*Interface origin of the e-ph anomalies*), the e-ph interaction due to the dynamic deformation potential acting on the interface space-charge electrons is shown to be responsible for the anomalies. The fact that the anomalies are removed by a magnetic field further supports the e-ph mechanism. The presence and concentration of space-charge electrons at the film-substrate interface is supported by a DFT band-structure calculations, in qualitative agreement with Hall measurements. In the third Section (*Calculation of the e-ph anomalies*), the model resulting from the above analysis is used to calculate the shape of the anomalies. The agreement between theory and experiment is presented as a strong argument in favour of the measurement of mode-selected e-ph interaction at film/substrate interfaces by use of BLS spectroscopy. The consequences of the present study, in particular a favourable comparison of present mode-selected e-ph coupling constants with those obtained with other methods in other spectral regions, are presented in the *Discussion and Conclusions*, while various experimental and theoretical details are collected in the Supplementary Material (*Suppl. Mat*).

## Results

### Brillouin light scattering data

**Figure 1:**
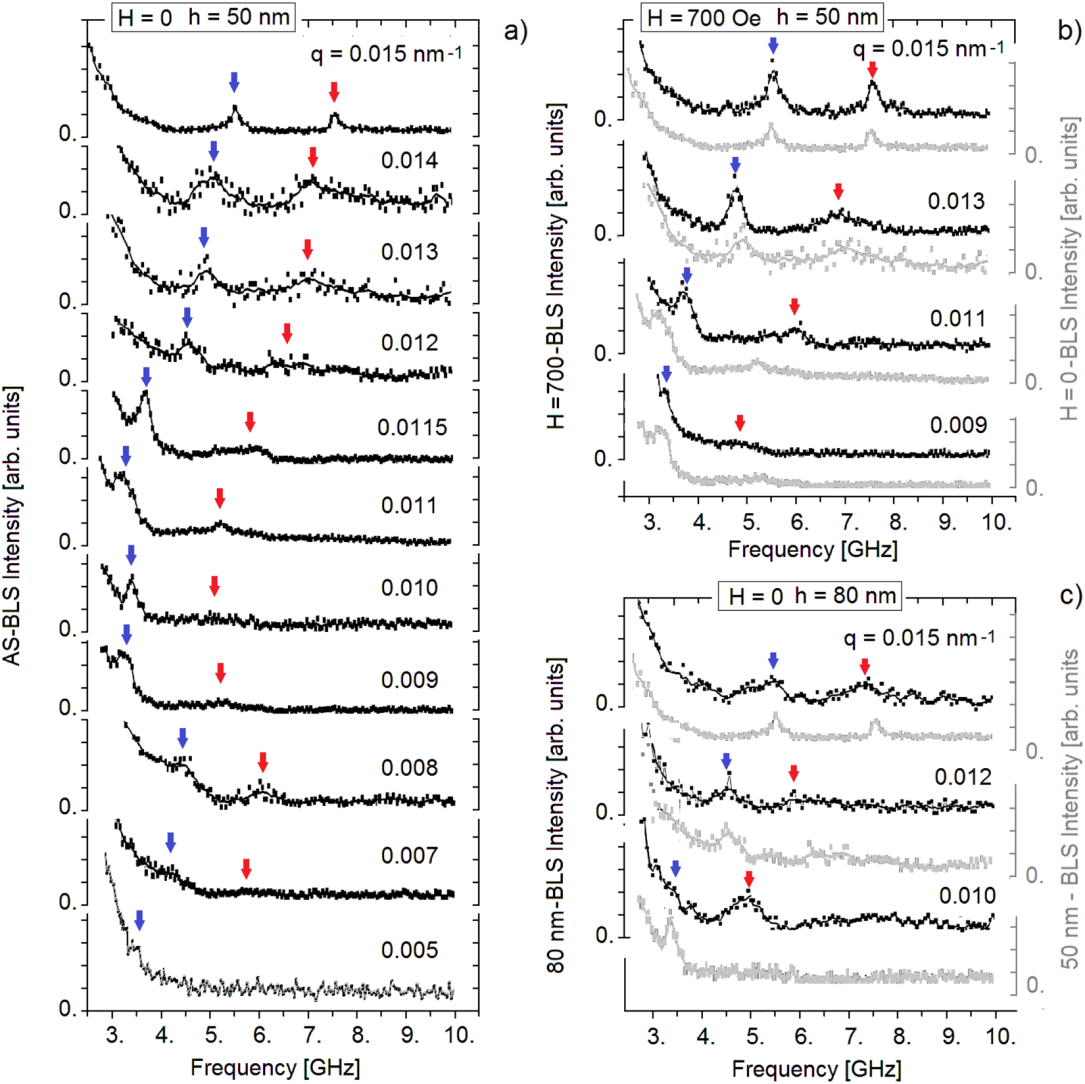
(**a**) A selection of BLS spectra for different values of the parallel wavevector transfer *q* for the highly-doped (*n*_ = 7.5 · 10^19^ cm^−3^) thinner (50 nm) film of Bi_2_Te_3_(111) on a GaAs substrate (sample A) with no external magnetic field. (**b**) A few spectra of sample A measured with an external magnetic field (700 Oe) applied normal to the surface (black dots) are compared with the corresponding spectra at *H* = 0 (gray dots). Similarly in panel (**c**), where a comparison is made between some spectra for the thicker low-doped sample B at *H* = 0 (black dots) with the corresponding spectra of sample A (gray dots). In all panels blue and red arrows indicate features attributed to RW and LR modes, respectively.

**Figure 2:**
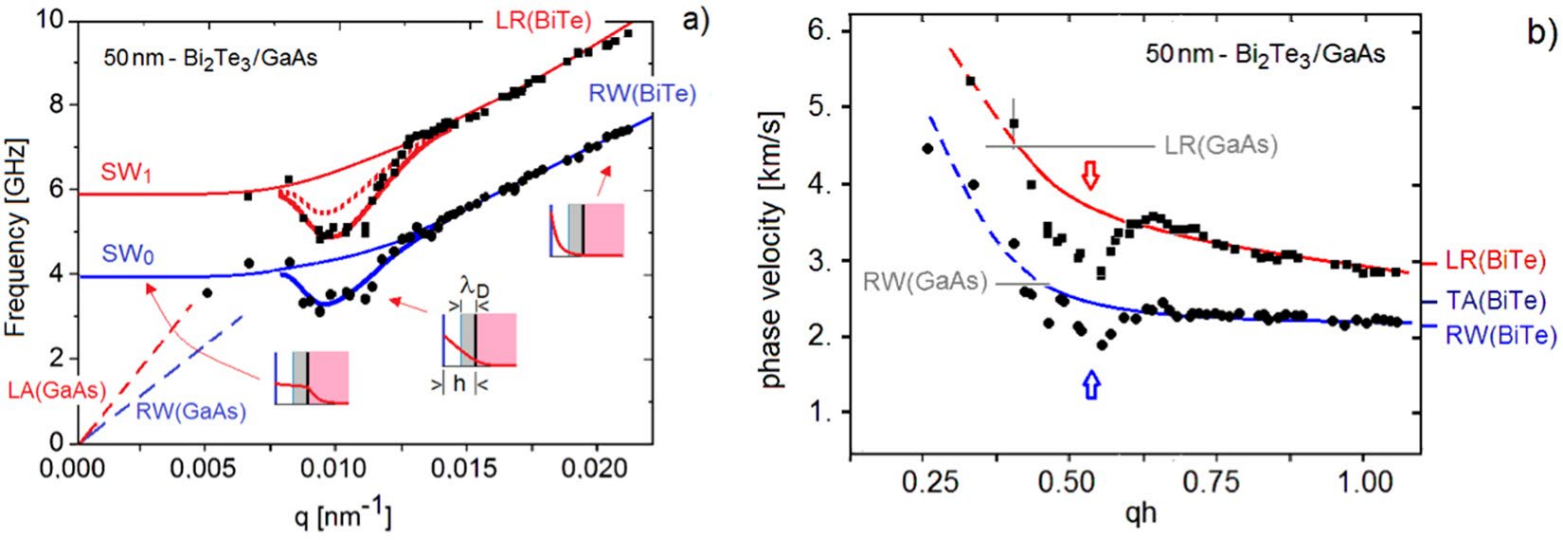
(**a**) The dispersion curves (thin lines) of the RW and LR branches of Bi_2_Te_3_(111) film, confined at the surface for large wavevectors *q*, turn smoothly into those of the corresponding leaky waves (SW_0,1_) for smaller *q*, when the corresponding penetration lengths would exceed the film thickness. The avoided crossings with the substrate RW and LR branches (broken lines) is here neglected in view of the weak substrate-film coupling. When the strong e-ph interaction in the interface space-charge region is considered, deep Kohn anomalies (thick and dotted lines) are produced, in agreement with the BLS data for the highly-doped 50 nm Bi_2_Te_3_(111) film. The insets explain the mechanism: anomalies occur when the penetration depth of the surface modes is comparable to the film thickness, producing a large displacement gradient in the space-charge region (grey area).The red broken curve corresponds to the fit when the oscillatory part in the LR eigenvector is neglected (see Eq. (7) and related text). (**b**) Phase velocity vs. wavevector times thickness. The experimental points deduced from (**a**) show the anomaly in the LR (red arrow) and RW (blue arrow) mode as compared with the expected fits. The corresponding LR and RW phase velocity in GaAs are marked with a straight gray line.

**Figure 3:**
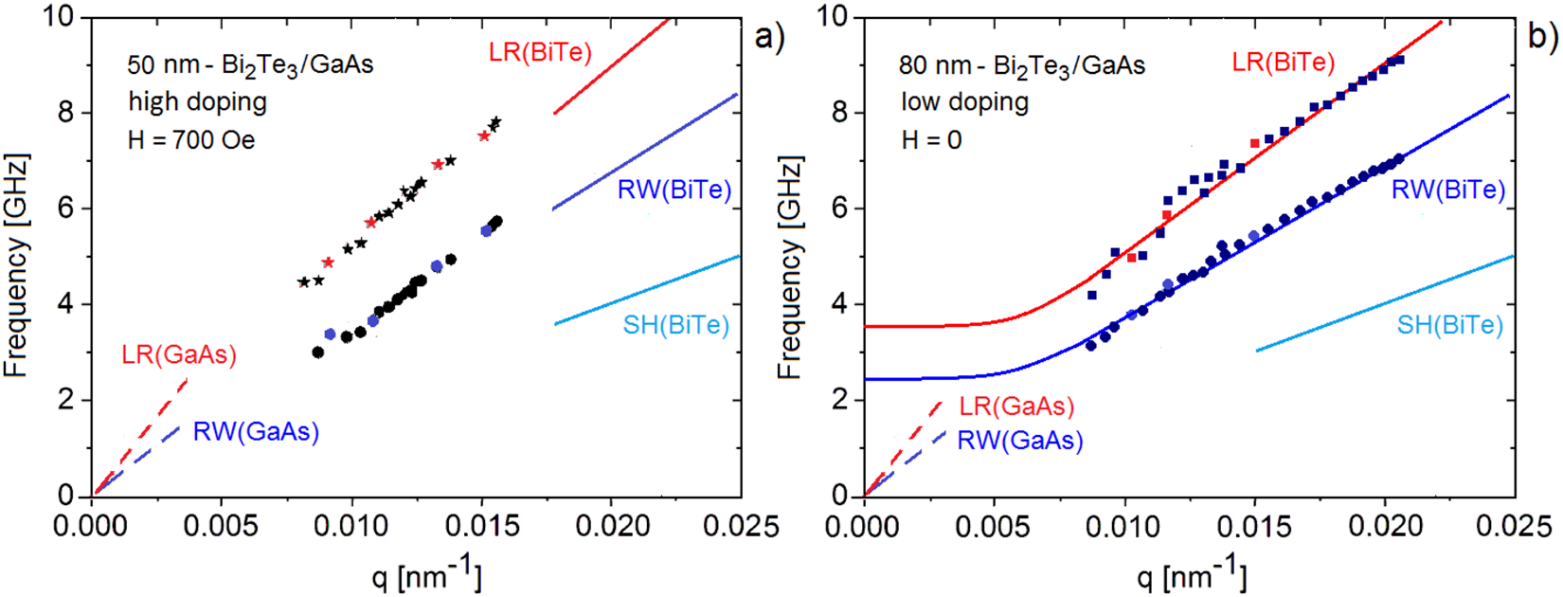
(**a**) The BLS dispersion curves of RWs and LRs of the highly-doped 50 nm film (sample A) under a 700 Oe magnetic field: the measured branches do not show any anomaly and are quite similar to those in panel (b) for the thicker low-doped film (sample B) in the absence of magnetic field. Data points in color correspond to the spectra shown in Fig. 1(b,c). In panel (a) the experimental data are compared with the slopes of the RW and LR branches of Bi_2_Te_3_(111) (full lines) and of GaAs(001) (broken lines), while in panel (b) they are compared with a fit for the supported film where the *q* = 0 frequencies are scaled down by a factor 50/80 with respect to those of Fig. 2. The plots also show the calculated anomalies (thicker lines), which fall just below the observation range of present experiments and are reduced in size to almost one half of those for the 50 nm sample A. The shear horizontal (SH) mode of Bi_2_Te_3_ is also drawn (light blue line) in order to show that the RW is actually a pseudo-surface mode^29^.

The samples of Bi_2_Te_3_ investigated in our experiments were grown by Molecular Beam Epitaxy (MBE) on a GaAs(001) substrate, actually oriented 2° off towards the [111] direction, which corresponds to the vicinal surface (1, 1, 41). Details of the preparation and measurements are given in the Section *Methods*. Hall effect measurements at room temperature allowed to determine the concentration and mobility of charge carriers in the two investigated samples. In sample A (thickness *h* = 50 nm), the carrier concentration is ${n}_{-}$ = 7.5 · 10^19^ cm^−3^, while in sample B (thickness *h* = 80 nm), ${n}_{-}$ = 1.6 · 10^19^ cm^−3^. Both samples have a similar mobility of 500 cm^2^/Vs. Figure 1 displays a series of BLS spectra for sample A as a function of frequency up to 10 GHz for different parallel wavevectors *q* in the range 0.005 ÷ 0.015 nm^−1^. Panels (a,c) report data in the absence of magnetic field while in panel (b) data were taken in presence of a magnetic field *H* = 700 Oe, orthogonal to the interface ($\widehat{z}$ direction). In order to show the modifications induced by the magnetic field or by the change of thickness, the spectra in panels (b) and (c) are compared with the corresponding spectra of sample A at *H* = 0 (light gray). The arrows in panel a) indicate the peaks corresponding to the RWs (blue arrows) and the weaker features associated with the longitudinal resonance (LR) (red arrows), the attributions being based on the correspondence with the RW and LR speeds derived from the experimental elastic constants of Bi_2_Te_3_^26^. Despite this close correspondence, the weaker peak can also be assigned to the next SW (SW_1_), as often found with BLS in supported thin films^27,28^. Since the assignment does not affect the general features of the following discussion, the label LR shall be used hereafter. In any case the above correspondence is well verified for the sharpest peaks observed at *q* = 0.015 nm^−1^ (panel (a)), while the peaks for decreasing values of *q* strongly deviate from the expected linear dispersion: both RW and LR frequencies exhibit a dip around *q* = 0.011 nm^−1^(see Fig. 2(a)). On the contrary the RW and LR frequencies for the *h* = 50 nm sample at *H* = 700 Oe (Fig. 1b)) and for the *h* = 80 nm sample at *H* = 0 (Fig. 1(c)) approximately show the expected linear dependence on *q* within the explored range (see Fig. 3(a,b)). The apparent removal of the dip by application of the magnetic field suggests an electronic origin of the anomaly.

The resulting dispersion curves are plotted in Fig. 2(a) for sample A at *H* = 0, in Fig. 3(a) for sample A at *H* = 700 Oe and in Fig. 3(b) for sample B at *H* = 0.The phase velocity of the two branches for sample A at *H* = 0 is plotted in Fig. 2(b) as a function of *q*. The large frequency dips observed in Fig. 2(a,b) are reminiscent of strong Kohn anomalies, while apparently no anomaly occurs in this range of *q* when the 700 Oe magnetic field is applied (Fig. 3(a)) nor in the low-doped thicker sample at *H* = 0 (Fig. 3(b)). The data are interpreted on the basis of a theoretical analysis described in the following sections and providing the dispersion curves (full lines) plotted in Figs 2 and 3.

A RW propagating with a wavevector *q* and a velocity *v*_*RW*_ along the surface of a semi-infinite solid decays exponentially with the distance *z* from the surface. At sufficiently large distance the dominant component of the normalized RW displacement field is polarized in the *z* direction and given by^22^

1 $${u}_{RW,z}(q,z)={(\frac{h\alpha }{{\mu }_{l}{\nu }_{RW}})}^{1/2}\exp (-\alpha qz),$$

where *μ*_*l*_ is the linear mass density in the direction normal to the surface averaged over the Bi_2_Te_3_ unit cell, and $\alpha ={(1-{\nu }_{RW}^{2}/{\nu }_{TA}^{2})}^{1/2}$ is the decay coefficient, with *v*_*TA*_ the transverse acoustic (TA) velocity of shear vertical (SV) polarization in the *q* direction. In the specific case of Bi_2_Te_3_(111), where *v*_*RW*_ = 2.09 km/s (Fig. 2) and *v*_*TA*_ = 2.19 km/s^24^, α ≅ 0.3.

For a film of thickness *h* deposited on a more rigid substrate, the RW is that of the film, denoted RW(BiTe) in Fig. 2, only for a penetration length much smaller than *h*, i.e., for $q\gg 1/ah$. For $q\ll 1/ah$ the penetration length would become much larger than the film thickness and, due to the confinement imposed by the larger substrate stiffness (and neglecting the avoided crossing with the RW of the substrate), the RW evolves into the lowest Sezawa wave of transverse polarization (SW_0_)^25^. Schematically the dispersion curve can be approximated by

2 $${\omega }_{RW}(q)={({\omega }_{0T}^{2}+{\nu }_{RW}^{2}{q}^{2})}^{1/2},$$

where ${\omega }_{0T}$ is the *q* = 0 limit of the film flexural angular frequency (*organ-pipe* frequency^24^).

The transition region, where the dispersion curve for decreasing *q* deviates from the linearity and bends towards a nearly constant frequency at small *q*, occurs where the penetration length is of the order of the film thickness and confinement starts being effective. When the avoided crossing with the substrate RW is considered (broken lines in Figs 2 and 3), the natural limit of the RW for *q* → 0 (diverging penetration length) is the substrate RW. For *q* increasing from 0, the penetration length decreases and the effects of the films start producing deviations from linearity already at penetration lengths considerably larger than *h*, due to the fact that the mass of Bi_2_Te_3_ layers is sizeably larger than that of GaAs (*loading effect*). This is one reason for observing the transition region around values of *q* which are quite smaller than 1/*αh*. Another reason is that ${\omega }_{0T}$ depends on film-substrate coupling and is softened for a softening of this coupling; in the extreme case of vanishing coupling, ${\omega }_{0T}$ → 0. Thus the actual value of ${\omega }_{0T}$ reflects both the interface interplanar force-constant softening and the loading effect. However for a given film density and substrate-film interaction, ${\omega }_{0T}$ is inversely proportional to *h*. The interplanar force constant softening also causes a faster decay of the RW in the interface region which may be accounted for by a local decay coefficient α* > *α*.

It should be noted that, strictly speaking, Bi_2_Te_3_(111) has no real RW in the *x-*direction, since the velocity of the *z*-polarized TA mode in this direction (2.19 km/s) is well above the velocity of the *y*-polarized TA mode, which is only 1.24 km/s (room temperature values from^24^, see Fig. 3). In this case the RW branch is embedded in the continuum band of the *y*-polarized TA modes: it is in general a resonance except in the symmetry direction where the polarizations of the RW and the band modes are exactly orthogonal. It is better known as “pseudo-surface wave”^22^, but hereafter it will however be labelled as RW.

The LR mode is not a macroscopic mode like the RW and only exists at finite wavevectors, its speed tending in the semi-infinite crystal, for *q* → 0, to that of the bulk longitudinal acoustic (LA) wave in the same direction. On the other hand at finite *q* it is localized with respect to the bulk LA band, falling however into the band of TA modes thus acquiring a broad resonance character. The displacement field has a dominant, exponentially decaying, LA component and an oscillatory TA component, which provide an elliptical polarization in the sagittal plane, similar (albeit orthogonal) to that of the RW. For a film on a more rigid substrate, also LR evolves for *q* → 0 into its own SW (SW_1_) with a *q* = 0 limiting frequency *ω*_0*L*_ also strongly dependent on the film-substrate interaction. Despite the broader and generally weaker signals from the LR mode, the large anomaly indicates an important e-ph interaction: this peculiarity of the ubiquitous LR in metal and semimetal surfaces has been established with HAS spectroscopy, where the LR intensity is often larger than RW’s^23,29^. Due to the deviation from linearity of decreasing *q*, the dispersion curve penetrates into the quasi-continuous spectrum of the bulk modes of the film and then into that of the substrate. Thus the SW_T_ mode is actually a resonance, with a finite lifetime and some dissipation into the substrate (leaky wave).

### Interface origin of electron-phonon anomalies

The following arguments support the attribution of the observed phonon anomalies to the e-ph coupling at the film-substrate interface. The electron deformation potential produced by a RW is proportional to its strain field ${\nabla }_{z}{u}_{RW,z}(q,z)$, and the softening of the RW frequency $h{\omega }_{RW}(q)$ caused by the electron response is, as shown below^30^, proportional to a matrix element between Fermi-level electronic states of ${|{\nabla }_{z}{u}_{RW,z}(q,z)|}^{2}/h{\omega }_{RW}(q)$. Thus the strongest e-ph softening occurs at the wavevector $q={q}_{{\rm{\max }}}$ where the latter expression is the largest. For the displacement field given by Eq. (1) and after substituting *α* with the local α* discussed above, it is ${q}_{{\rm{\max }}}=1/2{\alpha }^{\ast }h$, which for α* ~ 1 and *z* = *h* yields ${q}_{{\rm{\max }}}$ just in the observed range of 0.01 nm^−1^. This estimate, besides confirming the interface e-ph interaction as the cause for the observed anomalies, also suggests some softening of the interface force-constant (giving α* ~ 3*α*), as actually found below from DFT calculations. We remark that the strain field component ${\nabla }_{z}{u}_{RW,z}(q,z)$ responsible for a large e-ph interaction is the one which modulates the interplanar distances. In the present extreme acoustic limit, the interplanar distances which oscillate most are those between two adjacent QLs, whereas for the highest *z-*polarized optical phonons the modulation of the interlayer distances is mostly inside the QL. As recently shown by Monserrat and Vanderbilt^31^ and by Heid *et al*.^32^, the latter modes are actually those with the largest e-ph coupling. This can be understood from the fact that the bands around the bulk band gap of Bi_2_ Te_3_ are of p_z_ character^33^. This means that the electronic density is oriented along the stacking out-of-plane direction, and, as a consequence, the phonon modes that couple most strongly to these electronic states in Bi_2_Te_3_ are those that involve vibrations that modulate the interplane distance.

Similar arguments also hold true for the LR. The fact that the anomaly occurs in the transition region of the dispersion curves of both the RW and the LR means that the e-ph interaction responsible for the anomalies involves interface carriers. For the thicker (*h* = 80 nm) sample B the SW frequencies *ω*_0*T*_ and *ω*_0L_ are reduced by a factor 50/80 with respect to those of the thinner (*h* = 50 nm) sample A (Fig. 3(b)), and therefore the anomalies are predicted to fall slightly below the frequency range of present experiments.

The application of a magnetic field apparently removes the anomalies. This may be understood by considering that the cyclotron resonance frequency *eμ*′_0_ *H*/*m* ^*^ largely exceeds the anomalous phonon frequencies due to the small value of the carrier effective mass m*, thus inhibiting virtual phonon-induced electronic transitions at the Fermi level. A close inspection of Fig. 3a) shows however that the data points are aligned slightly above the theoretical RW and LR slopes, while the corresponding SW limit frequencies *ω*_0*T*_ and *ω*_0*L*_ are substantially smaller than in the absence of magnetic field. Thus the magnetic field seems to have some other exotic effect than just removing the anomalies, like, e.g., a further softening of the film-substrate interaction. Also this effect can in some way be related to the fact that the magnetic field applied in the $\widehat{z}$ direction favours the in-plane localization of the electron wavefunctions.

The thick curves providing a fitting of the anomalies in Fig. 2 result from the simple model for the e-ph interaction illustrated below. However, for a theoretical interpretation of the observed RW and LR dispersion curves and of the anomalies apparently induced by the e-ph interaction at the film/substrate interface we need first to model the interface and its electronic structure.

A brief comment on whether topologically protected states do occur at the film-substrate interface is in order.

In a Bi_2_Te_3_ film deposited onto Si(111), the Dirac cone dispersions of the topologically protected states at the two boundaries of the film start appearing when the thickness covers 2 to 4 quintuple layers (QLs), which means that, above this thickness, the electronic Dirac states at opposite boundaries do not overlap anymore and are therefore mostly localized at the surface and interface QLs^34–36^. In our case, as thoroughly discussed below, the Bi_2_Te_3_ film growth on GaAs(001) is preceded by the formation of a Te wetting layer passivating the dangling bonds, possibly followed by the formation of further Te buffer layers^37,38^. However, the actual structure of the substrate is not of great relevance, as long as the first substrate layer is made of Te planes because the Te work function (ranging from 4.76 eV for films^39^ to 4.95 for polycrystalline samples^40^ is comparable to that of intrinsic GaAs(001) (4.77 eV^41^) and both work functions are smaller than that of Bi_2_Te_3_(111) (5.30 eV^42^). This is important as the work function differences determine macroscopically the electron charge transfer from the substrate to the Bi_2_Te_3_ film.

**Figure 4:**
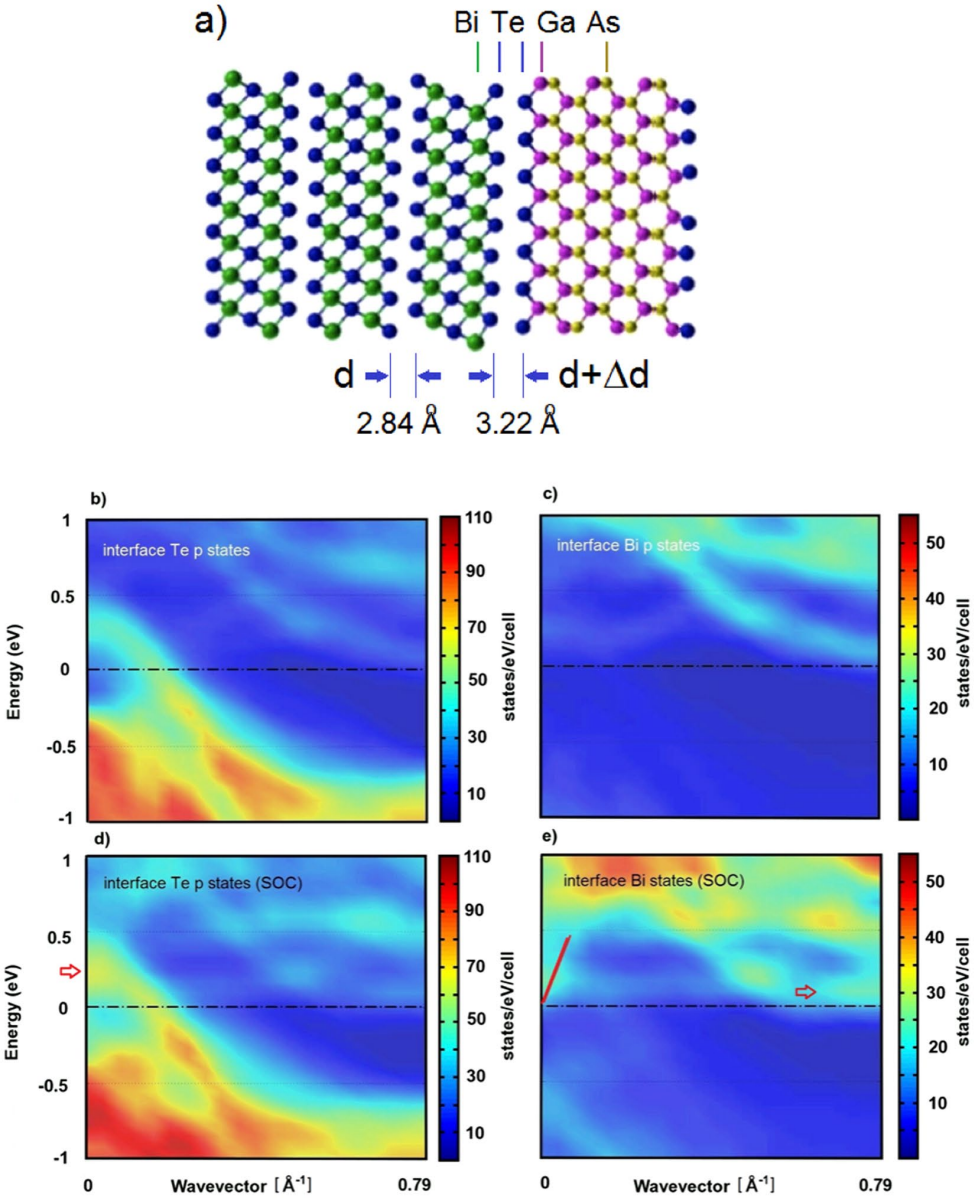
(**a**) Model of the Bi_2_Te_3_/Te/GaAs interface atomic structure used to calculate the interface DOS with DFT. Three Bi_2_Te_3_(111) quintuple layers on the l. h. side (Bi: green, Te: blue) are matched with a GaAs (001) substrate (Ga: lilac, As: yellow) passivated with a monolayer of Te atoms. The Te-Te interplanar distances are abtained from the DFT calculation including van der Waals potential and are in good agreement with Dycus *et al*. STEM data^38^. The hexagonal cell of Bi_2_Te_3_ spans 3 QLs with lattice parameters *a* = 4.38 Å and *c* = 30.48 Å. (**b**–**e**) Contour plots of the contributions to the electronic DOS projected onto the Bi_2_Te_3_/Te/GaAs interface projected on different atomic orbitals, as calculated without (**b**,**c**) and with (**d**,**e**) spin-orbit coupling (SOC). The electronic bands projected onto the interface Te p-states (**b**,**d**) and the interface (bottom QL) Bi states (**c**,**e**) are plotted as a function of the parallel wavevector along the surface symmetry direction = 0.79 Å^−1^), with the energy zero taken at the Fermi level. The red arrow in (**e**) marks a rather flat electron pocket extended near the zone edge. The red line in (**e**) highlights a linear energy dispersion that can be identified as the Dirac cone dispersion at the interface with the substrate.

With this in mind, the model depicted in Fig. 4(a) has been adopted for a DFT analysis of the interface. The structure is constituted by 3 QLs of Bi_2_Te_3_ on a GaAs(001) slab made of 6 Ga and 5 As planes plus 2 Te wetting layers saturating the Ga dangling at the two GaAs surfaces^43^. The DFT calculation provides the equilibrium configuration of the superlattice and its electronic band structure. It appears that the spacing between the two adjacent Te planes at the interface (3.22 Å, in agreement with 3.3 Å found in experiment, see Fig. S1(Suppl. Mat.) ^38^) is by more than 10% larger than that between two adjacent QLs in the bulk (2.84 Å, Δ*d* = 0.38 Å). The calculated interplanar distances as well as our fits of the phonon dispersions, suggest that there is a strong softening of the interface force constant^44–46^, occurring between the bottom QL Te plane and the adjacent substrate Te plane. This has an important consequence on the local dynamic strain induced by a lattice acoustic wave: the expected sizeable reduction of the interplanar force constant produces a corresponding enhancement in the dynamic strain with respect to that expected at the ideal interface between two homogeneous elastic media. This means a larger deformation potential and therefore a larger interface e-ph interaction.

The interplanar force constant softening is essentially that of the short-range repulsion, and is easily estimated as given by the factor $\exp (-{\rm{\Delta }}d/\rho )=0.263$, where *ρ* = 0.284 Å is the Born-Mayer Te-Te repulsive parameter^47^. The electronic bands, calculated over 30 points in the high-symmetry direction $\overline{{\rm{\Gamma }}M}$ of the Bi_2_Te_3_(111) Brillouin zone (BZ), are shown in Fig. 4(b–e) through the contour plots of the density-of-state (DOS) projected onto the atomic orbitals of interface atoms: panels (b,d) refer to Te p-states without and with spin-orbit coupling (SOC), respectively, and similarly panels (c,e) for the states of the Bi layer closest to the interface. The projection reveals that the metallic character is dominated by two main features. First, there is a band starting at 0.2 eV above the Fermi level (located at zero energy) at $\overline{{\rm{\Gamma }}}$ with a downward dispersion, so as to form a hole pocket mainly involving the Te p-states and strongly localized on the Te buffer layer. This is consistent with the fact that crystalline Te films behave as a small-gap p-type semiconductor^48^ and are used to cap TI surfaces thanks to their insulating properties^49^. In the present DFT result which includes just one single Te layer on GaAs, the Fermi level is seen to cut the Te hole band at about its inflection point, thus giving a very low hole mobility. The second important feature is the band, marked by a red arrow near the zone edge in (e), also present in the calculation without SOC (panel (c)), which is only 10 to 30 meV above the Fermi level. The latter is mostly associated with the Bi p states and is more delocalized in the Bi_2_Te_3_ slab. This band can be easily populated by electrons in greater number at room temperature, thus offering an explanation for the observed n-type conductivity. The comparison between the band structure with and without the spin-orbit coupling (SOC) permits to identify a Dirac cone (red line in e)), now just above the Fermi level in the vicinity of the $\overline{{\rm{\Gamma }}}$-point for the orbitals projected on the interface Bi states. This is consistent with the expectation that the topological protected states are preserved at a sufficiently inert interface. At room temperature it can host high-mobility electrons but we expect that their contribution to transport is marginal.

### Calculation of the electron-phonon anomalies

The dynamic deformation potential, which determines the e-ph interaction between the surface phonons and the free electrons of the interface space charge, is derived from the strain dependence of the interface double-layer capacity. The latter contains, within the respective space-charge screening lengths ${\lambda }_{m}$, the negative free-electron charge density per unit surface ${\sigma }_{-}\equiv -e{n}_{s-}{\lambda }_{-}$, with ${n}_{s-}$ the density at the interface of the space-charge transferred to Bi_2_Te_3_, and the corresponding low-mobility positive space-charge density ${\sigma }_{+}\equiv -{\sigma }_{-}$ in the substrate. As seen in Fig. S1 of the *Suppl. Mat*., the latter is concentrated within the Te passivating film, whether this is a monolayer as in the simulation, or a multilayer as occurring in present samples. For diluted carrier densities ${\lambda }_{m}$ may be taken in the Debye form, ${\lambda }_{m}={({\varepsilon }_{0m}{k}_{B}T/{n}_{sm}{e}^{2})}^{1/2}$ with ${\varepsilon }_{0m}$ the static dielectric permeabilities. However at carrier concentrations above 10^19^ cm^−3^, as in the present films where a few electronic bands cut the Fermi level (Fig. 4(d,e)) lending a degenerate character to the electron gas, the Thomas-Fermi form for screening length is more appropriate. This is simply obtained for the electron carrier (Bi_2_Te_3_) side by replacing *k*_*B*_ *T* in the Debye length expression with $\frac{3}{2}{\varepsilon }_{F}$, where ${\varepsilon }_{F}$ is the Fermi energy relative to the conduction band minimum^50^. The phonon-modulated potential is then written as

3 $${\rm{\Delta }}{V}_{q}(z)=-{\sigma }_{-}(\frac{{\lambda }_{-}}{{\varepsilon }_{0-}}+\frac{{\lambda }_{+}}{{\varepsilon }_{0+}}){{\rm{\nabla }}}_{z}{u}_{z}(q,h),\quad h-{z}_{-}\le z\le h+{z}_{+}$$

with the assumption that ${\rm{\Delta }}{V}_{q}(z)$ is zero outside the space-charge region and constant inside. The permeabilities of Bi_2_Te_3_ and Te are ${\varepsilon }_{0-}=290{\varepsilon }_{0}$ and ${\varepsilon }_{0+}=28{\varepsilon }_{0}$, respectively, where ${\varepsilon }_{0}$ is the vacuum permeability. On the basis of the recent accurate measurements of Hall mobility in polycrystalline p-Te as a function of temperature and carrier density^51^ it appears that at room temperature and given the high density of the hole space charge, the hole mobility is much smaller than the n-mobility of 500 cm^2^/Vs observed for the present Bi_2_Te_3_ films. Moreover the carrier concentration estimated from the DFT calculation (see *Suppl. Mat*.) indicates that the observed Hall conductivity can be essentially associated with the negative space charge within the Bi_2_Te_3_ QL at the interface. Only its contribution is considered in the following.

The matrix element ${h}_{ep}(q)$ of the phonon-modulated potential is then taken between the initial and final state electronic wavefunctions ${e}^{iKR}{\phi }_{Kn}(z)$ and ${e}^{-iK\text{'}R}{\phi }_{{K}^{{\rm{^{\prime} }}}{n}^{{\rm{^{\prime} }}}}^{\ast }(z)$ where **r** = (**R**, *z*) is the position, **K** and **K**′ the initial and final electron wavevectors, and *n, n*′ the labels of the film quantum-well states at the Fermi level. Plane waves are assumed for the motion parallel to the surface, while the motion normal to the surface is described by the wavefunctions ${\phi }_{Kn}(z)$. Thus

4 $$\begin{array}{ccc}{h}_{ep,q}(q) & = & e{\sum }_{{\bf{K}}n,{{\bf{K}}}^{{\rm{^{\prime} }}}{n}^{{\rm{^{\prime} }}}}^{\text{'}}{\delta }_{{{\bf{K}}}^{{\rm{^{\prime} }}},{\bf{K}}-(q,0)}\int dz{\phi }_{{{\bf{K}}}^{{\rm{^{\prime} }}}{n}^{{\rm{^{\prime} }}}}^{\ast }(z){\rm{\Delta }}{V}_{q}(z){\phi }_{{\bf{K}}n}(z)\\  & = & \eta {\varepsilon }_{F}{(\frac{2\hslash }{{\mu }_{l}\omega (q)})}^{1/2}{({\alpha }^{\ast }q)}^{3/2}\exp (-{\alpha }^{\ast }h\,q),\end{array}$$

5 $$\eta \equiv \frac{2}{3}{\sum }_{{\bf{K}}n,K^{\prime} n^{\prime} }^{({E}_{F})}{\delta }_{K^{\prime} ,K+(q,0)}{\int }_{sc}dz\,{\phi }_{{\bf{K}}^{\prime} n^{\prime} }^{\ast }(z)\,{\phi }_{{\bf{K}},n}(z)$$

where the sum is restricted to Fermi level states and the integral is over the space-charge (sc) region only. If the surface phonon wavevector *q*, which is here very small compared to the Fermi wavevector, is neglected in Eq. (5) and only intraband (*n* = *n*′) transitions are considered, the integral of Eq. (5) is ${\lambda }_{-}/h$. We note that only a few **K**, **K′** pairs on each Fermi contour fulfil parallel momentum conservation and the possible values of *n* = *n*′ are the number of conduction Quantum Well states at the Fermi level, which is of the order of the Bi planes (6 *h/c* with *c* = 3.45 nm the lattice constant in the *z* direction, encompassing 3 QLs). Thus *η* is of the order of a few units times $2{\lambda }_{-}/c$, the latter being guessed from the DFT calculation, (Fig. S1(Suppl. Mat.) (c)) to be of the order of 1/3. In what follows *η* is however treated as a fitting parameter.

The e-ph correction to the phonon energy, eventually yielding the anomaly, is then given by^52^:

6 $$\hslash {\rm{\Delta }}\omega (q)=-{h}_{ep,q}^{2}(q)/\hslash \omega (q).$$

This equation provides the minimum of the anomaly at ${q}_{{\rm{\max }}}\cong 1/(2{\alpha }^{\ast }h)$, which corresponds to ${\alpha }^{\ast }\cong 1$ to match with the experiment. With this value of α*, *μ*_*l*_ = 1.31 · 10^−14^ g/cm, ${\varepsilon }_{F}$ = 0.07 eV from the DFT calculation (Fig. S1(Suppl.Mat) (c)), and the RW unperturbed frequency of 4 GHz, it is readily found ${\rm{\Delta }}\omega ({q}_{{\rm{\max }}})/2\pi =-0.28{\eta }^{2}GHz$. The observed anomaly depth (~1 GHz) is reproduced with *η* ~ 2. When the same theory is applied to LR, the larger frequency yields, according to Eq. (4) a smaller anomaly (Fig. 2, dotted line), while the experiments shows instead a deeper anomaly. There is however an important difference between the RW eigenvector and that of the LR, which is a resonance superimposed to the bulk TA continuum. As such the z-component of the LR eigenvector has an oscillatory part which can be approximated as follows

7 $${u}_{LR,z}(q,z)=\frac{\gamma }{{(1+{\gamma }^{2})}^{1/2}}{(\frac{\hslash {\alpha }^{\ast }}{{\mu }_{l}{v}_{LA}})}^{1/2}{\rm{e}}{\rm{x}}{\rm{p}}(-{\alpha }^{\ast }qz+i{q}_{z}z),$$

where $\gamma ={\nu }_{LA}/{\nu }_{TA}=1.33$ ^26^ and ${q}_{z}=\gamma q$. When this is used in Eq. (4) with α* = 1, ${\rm{\Delta }}\omega ({q}_{{\rm{\max }}})/2\pi$ for the LR acquires the additional prefactor *γ* = 1.78, which leads to a good fit also for LR (Fig. 2, thick red line). A similar argument would also apply to SW_1_, if it is suggested as an alternative interpretation of the upper branch instead of LR, since its displacement field has one node in the z-direction and therefore an oscillating cosine factor^24^.

For the thicker (80 nm) sample B, the anomalies can be calculated in the same way. In this case, by assuming the same interface structure, the main effect is a shift of the anomalies to a smaller *q*_*max*_ = 0.0065 nm^−1^, just below the spectral range accessible to these experiments, with a 40% reduction in size (Fig. 3(b)).

As discussed above, an external magnetic field *H* orthogonal to the interface removes the anomalies (see Fig. 3(b)). By applying the field, the band structure evolves into Landau Levels (LLs), which mix the bands together. Moreover, the charge density wave in the space charge distribution provides also an oscillating in-plane component of the electric field *ε*_*q*_ which adds some dispersion to the LLs. Hence, interband contributions to Eq. (5) cannot be neglected. When the magnetic field is weak, we can adopt a semiclassical picture to estimate the change due to *B* in Eq. (5) by adding an interband correction to the Bloch wave functions ${e}^{iKR}{\phi }_{Kn}(z)$ ^53^. The overlap of the in-plane wave functions in the matrix element in Eqs (4, 5) is reduced, due to their incipient localization, as both *n* and **K** are no longer good quantum numbers. We estimate the anomaly reduction as $\delta {|{h}_{ep}(q)|}^{2}/h\omega (q)=-{q}^{2}{H}^{2}{\overline{{\rm{\Delta }}{V}_{q}}}^{2}/{\overline{{E}_{q}}}^{2}$, where ${\overline{{\rm{\Delta }}{V}_{q}}}^{2}$ is the average matrix element squared appearing in Eq. (3). This correction, can be understood as a drastic reduction of the parameter *η* in Eq. (5) [*see Suppl. Mat*.].

## Discussion and Conclusions

Brillouin Scattering in the GHz range of frequencies allows to explore the electronic properties of the interface with the GaAs substrate, by penetrating the Bi_2_Te_3_ film surface. The acoustic surface phonon modes, RW, and LR are clearly detected in a wavevector range two order of magnitude smaller than 2*k*_*F*_. It is not of a surprise that both RW, and LR display strong coupling to electrons. Indeed, both longitudinal and transverse components of the modes, have approximately comparable polarization in the z-direction when their penetration matches the film thickness (i.e. when *q* ~ 1/*ah*). For a rigid substrate the modes keep their polarization constant with respect to the 3D wavevector (*q*, 0, *π*/2 *h*). Since *α* and 2/*π* are comparable numbers, it appears that the modes, whether longitudinal or transverse with respect to the total wavevector, must have comparable (but not equal) *z-*components, when their penetration matches the film thickness. This conclusion holds approximately true when the substrate stiffness is finite, though much larger than that of the film.

While the complex interface with a heavy (Te) wetting layer introduces local dynamical perturbations so as to make the interface model between two homogeneous elastic media questionable, nevertheless the use of an effective *q* dependence of the decay length of the phonon mode appears to grasp the essential physics needed for the treatment of the anomalies, also keeping the treatment as simple as possible. As it appears from the fits in Fig. 2, the model provides the right position and shape of the anomalies, the depth being obtained by choosing the parameter *η* of about 2. When the low doping data (*n*_= 1.6·10^19^ cm^−3^) for the 80 nm thick film (sample B) are inserted into the same model, the anomalies are shifted to a smaller *q* (0.0065 nm^−1^) and are weaker (Fig. 3(b)). Measuring the shift of the anomaly as a function of the film thickness could be a further important test of the model.

The DFT analysis also permits to estimate an average carrier density of the same order of magnitude as derived from the Hall measurements (a few 10^19^ cm^−3^), with no difference between sample A and sample B. Indeed, the comparatively narrow anomalies can be exclusively associated with the interface space charge. It is likely that a substantial contribution to the carrier density originates in sample A from an extrinsic surface space charge due to surface defects, while it is negligible in sample B. However, it is important to remark in this respect that extrinsic carriers due to surface defects and trapped within the surface region would give a monotonous contribution to the e-ph interaction since the surface strain along *z* at *z* = 0 would grow linearly with wavevector *q* without producing any anomaly.

Finally, the mechanism by which an external magnetic field removes the anomalies in the high-doping case (Fig. 3(a)) can be understood as a drastic reduction of the parameter *η* in Eq. (5) due to the change of the in-plane matrix element of the e-ph interaction which follows from the formation of the Landau levels.

It is important to remark that the quasi 2D electron gas^54,55^ that plays a role here is the one localized at the interface within the Thomas-Fermi screening length, having in sample A a surface carrier density of about 10^12^ cm^−2^. However the charge transfer involving the Bi p-conduction band (Fig. 4(e)), with the Fermi level raised at the conduction band minimum by temperature, and a hole pocket due to Te bands localized at the interface, plays now the major role, thus making the presence of the interface Dirac cone insignificant. In any case, according to Das Sarma and Li^56^ a surface carrier density of 10^12^ cm^−2^ would give in a similar system like Bi_2_Se_3_ a *λ* of about 0.07 at 0 K and a smaller value at room temperature, to be compared with other estimates^43^. The observed anomaly dips allow to estimate the corresponding mode-selected e-ph coupling *λ*_*qv*_ from Allen and Dynes theory of superconductor phonon anomalies^57^ ${\lambda }_{q\nu }=3\overline{f}|{\rm{\Delta }}{\omega }_{q\nu }^{2}|/{\omega }_{q\nu }^{2}$. Here $\overline{f}$ is a constant that takes, for superconducting 6p metals and alloys, the best-fit value of 0.023. This gives *λ*_*qRW*_ = 0.060 and *λ*_*qLR*_ = 0.048, all consistent with the average $\lambda =\sum _{q\nu }{\lambda }_{q\nu }/3N$, where *N* is the number of atoms, *λ* = 0.05, as calculated by Huang^43^. As shown in^31^ and^32^, considerably larger values of *λ*_*qv*_ are found for the highest optical modes which strongly modulate the interplanar distances. It is important to remark that, while the size of the anomaly provides direct information on the e-ph coupling strength for a single phonon of selected energy and momentum, the position of the anomaly is basically a structural property and not a Fermi surface property.

In conclusion, interfaces can play an important role in future nanostructured devices, because they are protected from contamination^58^. In view of the great discrepancies in the evaluation of *λ* ^45,59–62^ at surfaces, it is of the great relevance to determine the mode-selected e-ph coupling strengths directly at the interfaces. We have shown that Brillouin Scattering offers a valuable tool to study the e-ph interaction at interfaces. The surface phonon modes excited by BLS act as a “*quantum sonar”* which probes the electronic properties of buried deep interface via e-ph coupling. The quantum sonar technique proposed here is similar to that recently used in HAS spectroscopy^29,30,63^, however with the roles of phonons and electron-hole pairs exchanged and very different spectral regions. BLS spectroscopy is suggested as a choice method for a non-invasive diagnostics in the GHz domain of e-ph interaction in transport properties of heterostructures and nano-electronic devices, at the interface of supported thin films.

## Methods

### Device Fabrication

Samples of Bi_2_Te_3_ investigated in our experiments were grown by Molecular Beam Epitaxy (MBE) on a GaAs (001) substrate, actually oriented 2° off towards the [111] direction, which corresponds to the vicinal surface (1,1,41). The terraces, oriented in the [110] direction, span therefore 41/√2 ≅ 28 lattice distances and can therefore accommodate exactly 4 interface (7 × 1) supercells (Figs 4(a) and S1(a)(Suppl.Mat.)). The oxides on the surface of the GaAs substrates were removed thermally in the growth chamber at 650 °C. Then the substrates were cooled down to 180 °C for the epitaxy. Prior to the growth, a 1 min Te soaking of the substrate surface was implemented in order to passivate the Ga and As surface dangling bonds^64^. Subsequently, the Bi source was opened together with Te one, to grow the Bi_2_Te_3_ thin film. The growth took place under ultra-high vacuum in the range of 10^−11^ Torr. The grown samples are *c* oriented, so that the surface is parallel to the quintuple layers of the Bi_2_Te_3_. The two materials combine through van der Waals (vdW) interactions that are considerably weaker than chemical bonds. Further details can be found in the *Suppl. Mat*.

### Brillouin Light Scattering spectroscopy

Surface elastic properties of Bi_2_Te_3_ were studied at room temperature with the use of a tandem type Brillouin spectrometer (JRS Scientific Instruments), ensuring the contrast of 10^10^ ^53^. A detailed description of the experimental setup can be found in^65–68^. The light source used was an Nd:YAG single-mode diode-pumped laser of power 200 mW, emitting the second harmonics of the length *λ*_*0*_ = 532 nm (Excelsior Spectra Physics). The light impinging on the sample was polarized in the sagittal plane defined by the normal to the sample surface and by the wave vector of a given phonon. Measurements were made in the backscattering geometry 180°. The wave vector of the phonons *q* could be varied from 1.1 · 10^−3^ nm^−1^ to 23.3 · 10^–3^ nm^−1^. The spectra accumulation time was 0.5 h.

### DFT simulations

The calculations were performed using density functional theory (DFT), as implemented in the QUANTUM-ESPRESSO package^69^, using norm conserving pseudopotentials and the local density approximation (LDA)^70^ for the exchange-correlation energy functional both including and neglecting the effect of spin-orbit coupling (SOC) treated in a non-collinear formalism. Semiempirical vdW interaction according to Grimme has been added^71^. The Bi_2_Te_3_(001)/GaAs(001) interface was modelled with a superlattice of 15 Bi_2_Te_3_ layers and 11 GaAs layers with both the GaAs surfaces passivated with a Te buffer layer. A supercell with in-plane lattice constants of 0.399 and 2.77 nm allowed to adjust a (4 × 1) supercell created starting from the rectangular non elementary supercell of Bi_2_Te_3_ on a (7 × 1) GaAs(001) supercell with a mismatch below 1.5%, as shown in Fig. 4(a). The model is constituted by three QLs of Bi_2_Te_3_ (as for its hexagonal (0001) representation) on a GaAs(001) slab made of six Ga and five As planes plus two Te wetting layers saturating the Ga dangling bonds at the two GaAs surfaces. Cyclic boundary conditions lead then to a Bi_2_Te_3_(0001)/Te/GaAs(001)/Te superlattice with the *x*-axis in common and two alike interfaces. Since the small-angle vicinal surface of GaAs occurring in experiment would expose (001) terraces with either Ga or As terminations, Te passivation has been considered for both cases, but that of Ga bonds turned out to be energetically more favourable and has been adopted. The electronic wave functions were expanded in plane waves up to a 45 Ry energy cut-off. The integration over the Brillouin Zone (BZ) was performed using an unshifted 6 × 1 × 1 Monkhorst-Pack mesh^72^. A gaussian smearing of 0.01 Ry was used. Atomic positions were relaxed until the forces were below a 0.5 · 10^−3^ a.u. threshold.

### Data availability statement

Data from BLS measurements are available upon request to M.Wiesner, mwiesner@amu.edu.pl. DFT and model data are available upon request to A.Tagliacozzo, arturo@na.infn.it.

## Electronic supplementary material

Supplementary Information
