# Supplementary material for: The electron-phonon interaction at deep Bi2Te3-semiconductor interfaces from Brillouin light scattering
Source: Sci Rep. 2017 Nov 27;7:16449. doi: 10.1038/s41598-017-16313-5 (PMC5703879; doi:10.1038/s41598-017-16313-5)
Supplement: Supplementary file 1 — Supplementary Information [file 41598_2017_16313_MOESM1_ESM.pdf]

SUPPLEMENTARY MATERIAL to:

The electron-phonon interaction at deep Bi_2Te_3-semiconductor interfaces from Brillouin light scattering

M.Wiesner[1,2], A. Trzaskowska[1], B.Mroz[1], S.Charpentier[3], S.Wang[4],Y.Song[3,4], F. Lombardi [3], P.Lucignano[5,6], G.Benedek[7], D.Campi[8], M.Bernasconi[8], F.Guinea[9,10] and A.Tagliacozzo[6,5,11,*]

1. Faculty of Physics, Adam Mickiewicz University, Umultowska 85, PL61614 Poznan, Poland,

2. The NanoBioMedical Centre, Adam Mickiewicz University, Umultowska 85, PL 61614 Poznan, Poland,

3. Department of Microtechnology and Nanoscience, Chalmers University of Technology, SE-412 96 Göteborg, Sweden,

4. State Key Laboratory of Functional Materials for Informatics, Shanghai Institute of Microsystem and Information Technology, Chinese Academy of Sciences, 865 Changning Road, Shanghai 200050, China,

5. CNR-SPIN, Monte S. Angelo-Via Cintia, I-80126, Napoli, Italy ,

6. Dip. di Fisica, Università di Napoli Federico II, Via Cintia, I-80126 Napoli, Italy,

7. Donostia International Physics Centre (DIPC), Paseo Manuel de Lardizábal 4, 20018 Donostia/San Sebastian, Spain,

8. Dipartimento di Scienza dei Materiali, Università di Milano-Bicocca, Via Cozzi 53, 20125 Milano, Italy,

9. Imdea Nanociencia, Faraday 9, Cantoblanco, 28049 Madrid, Spain,

10. School of Physics and Astronomy, University of Manchester, Manchester M13 9PL, UK,

11. INFN, Laboratori Nazionali di Frascati, Via E.Fermi, Frascati (Italy).

**arturo@na.infn.it*

Information on the elastic waves propagating in supported films may be found in the book edited by C. L. Muhlstein and S. B. Brown, Mechanical Properties of Structural Films (American Society for Testing and Materials (ASTM), Bridgeport 2001). See particularly the article by R. Pastorelli, S. Tarantola, M. G. Beghi, C, E, Bottani, and A. Saltelli, p. 152-167. In this chapter also the LR branch is discussed.

The studies of the dependence of the BLS intensity and frequency shift of Surface Acoustic Wave on the film thickness [S1] show that the TI thickness of 50-60 nm is optimal for BLS. In the case of a thin layer on thick substrate: a. If the layer is too thin substrate's contribution to a recorded spectrum dominates and phonons related to the thin layer are hardly visible. b. If the layer is too thick the observed phonons show more bulk behaviour.

Moreover, the intensity of observed peaks for selected thickness of the thin layer strongly depends on the angle. This means that for small thickness of the layer the dispersion relation is difficult to measure (in narrow angle range). So again thickness of 50 nm for TI was optimal for BLS.

Sample growth [S2][37]

A Riber Compact21 MBE system was employed for the Bi_2Te_3 thin film growth. The sample R0680 was grown on an insulating Si(111) substrate and the R0715 on a vicinal semi-insulating GaAs(001) substrate with a 2°offcut towards [110]. Before the growth, the Si substrate was etched by a mixed chemical solution of HF and NH_2F to remove the surface oxide, and then quickly transferred to the degas chamber of the MBE and heated to 600 °C until the pressure in the chamber dropped below 1×10^{-8} Torr. The GaAs substrate was deoxidated thermally in the growth chamber at 650 °C. then, the substrate temperature was set to 180°C and 190°C for R0715 and R0680, respectively, and a 1 min Te soaking was implemented to passivate the surface dangling bonds. The Te flux was selected to be below the vapor pressure at the selected growth temperature to ensure no significant Te accumulation. Then the Bi and Te atoms were incident to the substrate to grow the Bi_2Te_3 thin films. For both samples, the beam equivalent pressure (BEP) of Te and Bi was 1.0E-6 Torr and 1.0E-8 Torr, respectively, resulting in a growth rate of 50 nm/ h. For the sample R0680, a Bi_2Te_3 seed layer was firstly grown for 4 mins at 180°C, then the growth temperature was linearly increased to 220°C with a gradient of 15°C/min without growth stop. The growth temperature was kept 220°C for the rest of the thin film and the total thickness was 75 nm. For the sample R0715, the growth temperature was 180°C constantly. While, a growth procedure of "quintuple layer (QL) by QL" was employed, in which a 0.5 QL Bi_2Te_3 was grown followed by a 30s Te soaking and repeated for 100 times resulting in totally 50 nm. All the growth temperatures reported here are read by a thermocouple.

The growth front was *in situ* monitored by reflection high-energy electron diffraction (RHEED) during the growth. The Bi_2Te_3 1×1 pattern emerges immediately when the growth started.

DFT study of the electronic charge transfer at the interface.

The assumption that the observed effects of the e-ph interaction are related to a substantial amount of space-charge electrons transferred from the GaAs substrate to the Bi_2Te_3 has been tested with a DFT calculation of the electronic density of states (DOS) projected onto the interface, in the reduced layer structure sketched in Fig.4(a) of the main text. The structure consists of a superlattice with three quintuple layers of Bi_2Te_3 and eleven layers of the GaAs substrate. A Te wetting layer has been added at the interface, to mimick the heavy Te buffer layer introduced in the preparation of the samples to passivate the dangling bonds of the foremost substrate layer. This model is based on the image obtained by Dycus *et al* [S2] with atomic-resolution high-angle annular dark-field (HAADF) scanning transmission electron microscopy (STEM) reproduced in Fig. S1(b) as well as on the TEM image shown in (a) for the Bi_2Te_3(111)/Te/GaAs(001) interface. Whether the termination involves Ga or As dangling bonds has been decided by a preliminary DFT calculation comparing both options: the passivation of Ga bonds turned out to be energetically more favorable than that of As dangling bonds and was therefore adopted in the present analysis. The band structure for the Ga terminated surface, calculated over 30 points along the high symmetry ΓM direction of Bi_2Te_3(111) with and without spin-orbit coupling are shown in Fig.4(b-e). The projections on the atomic orbitals of the atoms closest to the interface reveal their metallic character dominated by two main features: i) a band starting at 0.2 eV above the Fermi level at the Γ-point and dispersing downward forming a small hole pocket mainly involving the Te p-states and

strongly localized on the Te buffer layer ii) a rather flat band lying from 10 to 30 meV above the Fermi level near the zone border, rather localized in the Bi_2Te_3 slab, that can be attributed to the Bi *p* states. This band can be easily populated by electrons in greater number at room temperature, with respect to the holes associated with the Te band, thus offering an explanation for the n-type conductivity. The comparison between the band structures with and without the spin-orbit coupling (SOC) suggests the identification of the linearly dispersing feature which appears in the vicinity of the Γ-point with a Dirac cone [red line in Fig. 4(e)]. However, the overlap with delocalised states close to Γ makes it difficult to determine its position with respect to the Fermi level.

The dependence of the charge transfer at the interface coming from the Te-*p*-orbitals of the wetting layer on the termination of GaAs stems from the space dependence of electrostatic potential across the interface. In the present case of a substrate surface oriented 2º away from [001] towards the [111] direction, both As- and Ga-exposed surfaces may occur, though Ga-Te bond appears to be favoured as said above, and in agreement with previous pseudo-potential calculations [S3], although metal-organic chemical vapour deposition experiments would indicate that Te is unable to passivate GaAs(001) [S4]. However low-resolution TEM pictures of other samples grown as the present ones indicate however that several layers of Te at the interface may be grown before the stoichiometric growth of the Bi_2Te_3. In other words the Te is not just saturating the dangling bonds of the GaAs but may also grow on the substrate in the form of a thin film.

In Fig.S1(c) we report the calculated electrostatic potential energy for the electrons of the Bi_2Te_3-Te-GaAs-Te superlattice. The averaged electrostatic potential energy (*green curve*) reveals a net dipole from the bulk GaAs towards the Bi_2Te_3. Electrons from the GaAs concentrate at the interface with the Te wetting layer, where the potential energy is lower. The electron potential energy calculation is compared with the HAADF-STEM profile of the Bi_2Te_3(111)/Te/GaAs(001) interface reported by Dycus et al [S3] (Fig S1(b)) who could assign the passivating layer to Te by means of atomic –resolution energy dispersive X-ray spectroscopy (EDS). Note that the HAADF-STEM profile resolves a single row of atoms in the z direction, whereas the electron potential energy profile is mediated in the *xy* plane. This explains the difference in the profiles for GaAs, the HAADF detecting GaAs every second bilayer, while the xy-plane average of the electron potential energy profile shows all GaAs bilayers. The profile of the DFT potential energy mediated in the *xy* plane (green line in Fig S1(c)) allows for an estimation of the average carrier concentration within the thickness *h* of the film from the equation $n_- = \varepsilon_{0-} V_- / (\lambda_- h e^2)$, where (Fig S1(c)) $V_- \cong 0.09$ eV, $\lambda_- \cong 1$ nm, and $\varepsilon_{0-} = 290\varepsilon_0$. For h = 50 nm and 80 nm, it is found $n_- \cong 3.9$ and $2.4\cdot10^{19}$ cm^{-3}, respectively. It is therefore concluded that a large fraction of the measured carrier densities constitutes the interface free-carrier space charge. The DFT calculation gives a Fermi level pinned at the bottom of the Bi(p) conduction band, the free-carrier space charge is mostly associated with the Te(p) bands which cut the Fermi level at about 2.5 Å^{-1} (cfr. Fig. 4(e) of main text) and localize the electrons in the pocket around z = 50 nm in Fig. S1(c); thus ε_F in Eq. (4) can be identified with V_-. The fraction of the measured n_- which is not at the interface is likely to accumulate at the surface populating the Dirac states and the quantum well minibands within the defect-induced band bending [S5].

We note that the present Hall carrier densities are in excellent agreement with those measured by Suh *et al* [S5] on BiTe films grown on GaAs at the measured carrier densities at the thicknesses of 80 and 50 nm. The increase of the free carrier density for decreasing thickness described in [S5] has been associated with the increasing weight of surface quantum well states

with respect to those of the bulk conduction band. Our work shows that this is perfectly sound, adding however the important information that a large, if not dominant, fraction of such quantum well states are at the interface. It is here important to mention that the surface free-carriers will also contribute to the e-ph interaction, but the effect on the RW and L mode is a smooth function of q which could be significant only at q values whose corresponding penetration length becomes shorter than the surface Thomas-Fermi screening length (~10 nm [S6]), thus completely outside the range explored in the present experiment. All this confirms that the observed anomalies can uniquely be associated with the free carriers at the film/substrate interface.

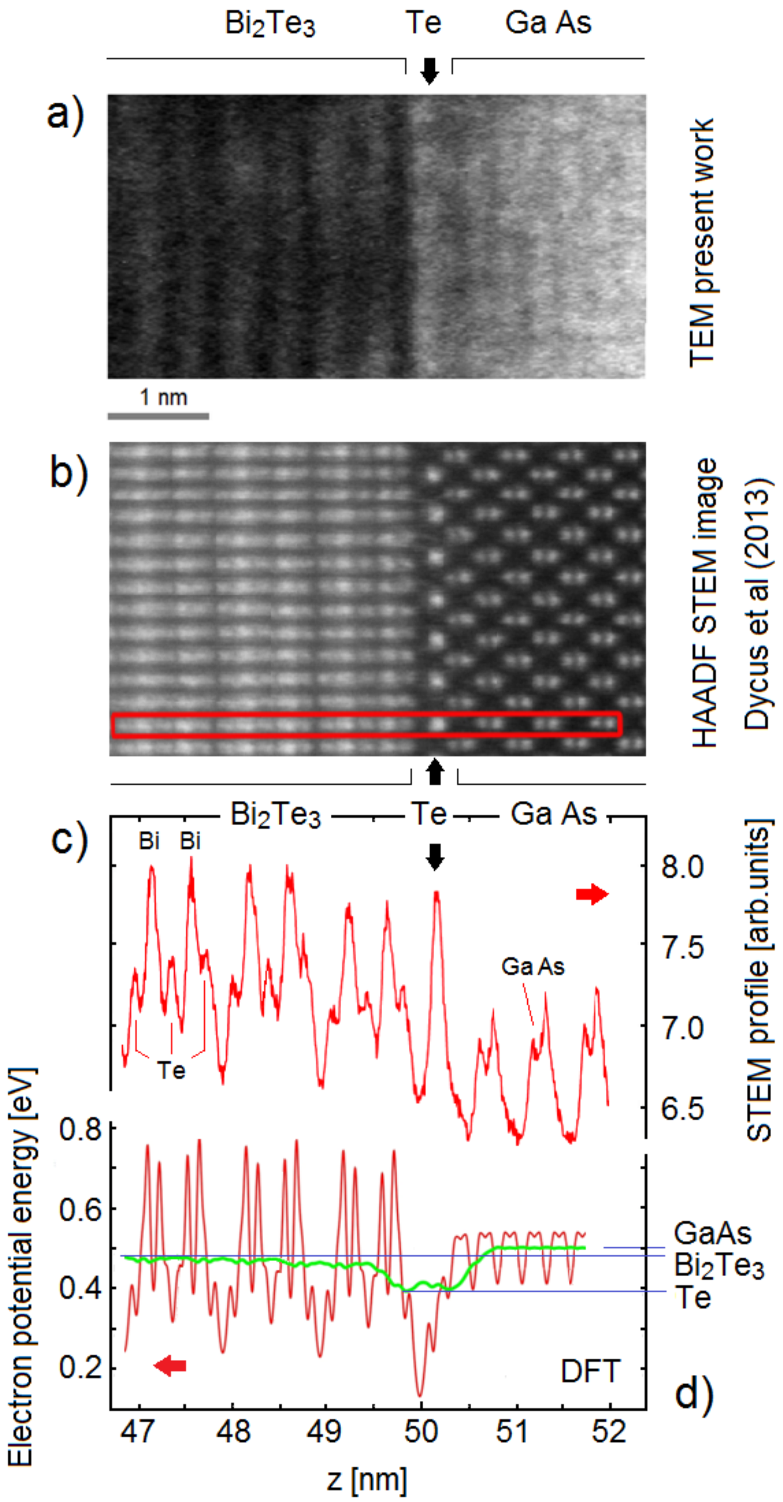

Figure S1. (a) Low-resolution TEM image of a Bi_2Te_3(111)/Te/GaAs(001) sample R0715 studied with BLS in the present work. This is compared with atomic-resolution high-angle annular dark-field (HAADF) scanning transmission electron microscopy (STEM) image (b), from Dycus *et al* [S2][37] and with the corresponding STEM profile (c) of the Bi_2Te_3(111)/Te/GaAs(001) interface. The comparison clearly confirms the presence of a passivating layer between the Bi_2Te_3 film and the GaAs substrate (vertical arrows), assigned by Dycus *et al* to Te by means of atomic–resolution energy dispersive X-ray spectroscopy (EDS) [S2]. In panel (d) the electron potential energy calculated with DFT is displayed and compared to the HAADF-STEM profile shown in panel (c) (red curve in (d)). The green line reports the electron potential energy averaged over the *xy*-plane. The average interface potential drop in the Bi_2Te_3 space charge region is $V_- \cong 0.09$ eV over an estimated thickness of about one QL ($\lambda_- \cong 1$ nm). The HAADF-STEM image of panel (b) has been adopted as a model of the Bi_2Te_3(111)/Te/GaAs(001) interface for present DFT simulations. Note that the HAADF-STEM profile, resolves a single row of atoms in the z direction (red horizontal rectangle in (b)). This explains the difference in the profiles for GaAs between panel (c) and (d): the HAADF detects GaAs every second bilayer, while the xy-plane average of the electron potential energy profile in (d) shows all GaAs bilayers.

Effects of the magnetic field

The mechanism by which an external magnetic field $B || \hat{z}$, orthogonal to the interface, removes the anomalies in the high-doping case (Fig. 3 a)) can be understood as a drastic reduction of the in-plane overlap of the wave functions $e^{i\,\mathbf{K}\cdot\boldsymbol{\rho}} u_{\mathbf{n}\,\mathbf{K}}(\varrho)$, induced by the incipient localization due to the formation of Landau levels (LLs). This appears as a reduction of the parameter η in Eq. (5) of the text. When the magnetic field is weak we can adopt a semiclassical picture to estimate the change due to B in Eq.(5,6). Semiclassical circular orbits have energy corresponding to the LL energies:

$$\frac{\hbar^2 k_i^2}{2m^*} = \left(i + \frac{1}{2}\right) \hbar\omega_c \ ,$$

where ω_c= eB/$m^* c$ is the cyclotron frequency and i is an integer. We consider a gauge in which particles are localized in the $\hat{x}$ direction. Addition of a vector potential $\vec{A}(\rho, 0) = (0, Bx, 0)$ requires the substitution $k_y \rightarrow k_y + eBx/c$. The δ−function in Eq. (4) is smeared in the $\hat{k}_x$ direction to account for the fact that the states are no longer eigenstates of k_x . Two circular semiclassical orbits having minimal separation in energy differ in area in k space by $\Delta(\pi k_i^2) = 2\pi eB/\hbar c$ $(\equiv s^2)$. k_x is no longer sharp because of the spreading of the area in k space. As $q \parallel \hat{x}$, a minimal spread in k_x can be introduced in the representation of the δ− function of Eq.(4), by using a gaussian of dispersion width s^2 and a form factor $g_{n'\,n}(k_x{}', k_x)$:

$$\eta\, h_{ep,n}(q) \cong \sum\nolimits'_{k_y, k_y{}'} \delta(k_y - k_y{}') \qquad \text{(S1)}$$

$$\times \sum\nolimits'_{k_x, k_x{}'} \frac{1}{s\sqrt{\pi}} e^{-\frac{(k_x - k'_x - q)^2}{s^2}} \sum\nolimits_{n'} g_{n'n}(\mathbf{K}', \mathbf{K}) \int dz\, \chi^*_{\mathbf{n}'\,\mathbf{K}'}(z)\, \Delta V_q(z)\, \chi_{\mathbf{n}\,\mathbf{K}}(z) \ ,$$

Here $\mathbf{K}', \mathbf{K}$ have the k_y components fixed equal by the δ-function. The magnetic field introduces a coupling between the bands so that the perturbed functions $u_{\boldsymbol{nK}}(\rho)$ are no longer orthogonal between different bands [S7]:

$$g_{n'n}(\mathbf{K}', \mathbf{K}) = \int d\rho\, u^*_{\boldsymbol{n}'\boldsymbol{K}'}(\rho)\, u_{\boldsymbol{nK}}(\rho). \qquad \text{(S2)}$$

The sum over n' is already included. In fact, as there is an electric field $\mathcal{E}_q$ in the space charge region, the indices n, n' are no longer independent of $k_y, k_y{}'$ according to the fact that the LLs acquire a dispersion themselves. In fact, in an adiabatic approximation, one could write:

$$E_{i\mathrm{K}} \cong \hbar\omega_c \left(i + \frac{1}{2} + \frac{e\mathcal{E}_q \ell_B^2 k_y}{\hbar\omega_c} \right) .$$

Here $\ell_B{}^2 = \hbar c / eB$ is the cyclotron radius. We concentrate on the terms with $\boldsymbol{n}' \neq \boldsymbol{n}$ which contribute in reducing the result of Eq.(S1) as we recognize that, by Schwarz inequality, is $|\sum_{\boldsymbol{n}'} g_{n'n}(\boldsymbol{K}', \boldsymbol{K})|^2 \leq 1$.

To estimate the form factors in Eq.(S2) we use the approximate expansion of the Bloch functions [S8]. For small q vectors the $k \cdot p$ approximation can be used. To lowest order, if $\vec{v}$ is the velocity in the band, we have:

$$u_{\boldsymbol{nK}'}(\vec{r}) = u_{\boldsymbol{nK}}(\vec{r}) + \sum_{\boldsymbol{n}' (\boldsymbol{n}' \neq \boldsymbol{n})} u_{\boldsymbol{n}'\boldsymbol{K}}(\vec{r}) \frac{\hbar(\boldsymbol{K}' - \boldsymbol{K})}{E_{n'k} - E_{nk}} \cdot$$

$$\cdot \int d\vec{r}\, u^*_{\boldsymbol{n}'\boldsymbol{K}}(\vec{r})\, e^{-i\boldsymbol{K}\cdot\vec{r}}\, \vec{v}\, e^{\,i\boldsymbol{K}\cdot\vec{r}} u_{\boldsymbol{nK}}(\vec{r}), \qquad \text{(S3)}$$

Corrections due to the normalization of $u_{\boldsymbol{nK}'}(\vec{r})$ are of order $O(|\boldsymbol{K}' - \boldsymbol{K}|^2)$ and will be neglected. Inserting Eq.(S3) into Eq.(S2) and using the minimal energy difference in the denominator, the approximated result for the the form factor contribution is:

$$|\, g_{n'n}(\boldsymbol{K}', \boldsymbol{K})|^2 = \begin{cases} 1 + O(|\boldsymbol{K}' - \boldsymbol{K}|^4) & for \quad n' = n \\ |\boldsymbol{K}' - \boldsymbol{K}|^2 \dfrac{\hbar^2}{2m} \dfrac{|1 - m/m^*|}{\hbar eB/m^* c}, & for \quad n' \neq n \end{cases}$$

where the $f - sum\ rule$ has been used [S8].

Performing the integral over $k_x{}' - k_x$ in Eq.(S1) and transforming the sum over k_y, n' into an integral over energies:

$$\sum_{k_y n'} \Rightarrow \frac{L}{2\pi} \int \frac{d\epsilon'}{\hbar v_D},$$

where $v_D = c\mathcal{E}_q / B$ is the drift velocity, we get from the term with $\boldsymbol{n}' \neq \boldsymbol{n}$:

$$\delta\left|\eta\, h_{ep,n}\,(q)\right|^2/\hbar\omega_c \cong -\left(\frac{L}{2\pi}\right)^2 \frac{\omega_c}{v_D}\left(\frac{q\ell_B}{\sqrt{2}}\right)^2 \left|\frac{m^*}{m}-1\right| \frac{1}{\hbar v_D}\left|\int dz\ \mathrm{e}\Delta V_q(z)\,|\,\chi_{\mathbf{n}\,\mathbf{0}}(z)|^2\right|^2. \tag{S4}$$

It follows that the correction per unit area is $\propto -\left(q^2B^2/\,\overline{\mathcal{E}_q{}^2}\right)\cdot\overline{\Delta V_q}^2$. Here $\overline{\Delta V_q}^2$ is the matrix element squared appearing in Eq.(S4).

REFERENCES

[S1] Every, A.G., Pang,W., Comins, J.D. & Stoddart, P.R., "Brillouin scattering study of guided modes in TiN films on high-speed steel", Ultrasonics 36, 223-227 (1998).

[S2] Dycus, J. H., White, R. M., Pierce, J. M., Venkatasubramanian, R. & LeBeau, "Atomic scale structure and chemistry of Bi[sub 2]Te[sub 3]/GaAs interfaces grown by metallorganic van der Waals epitaxy," J. M., Appl. Phys. Lett. 102, 081601 (2013)

[S3] Takahisa, O., "Passivation of GaAs(001) surfaces by chalcogen atoms (S, Se and Te)", Surf. Sci. 255, 229-236 (1991)

[S4] Chambers, S. A. & Sundaram, V. S., "Passivation of GaAs(001) surfaces by incorporation of group VI atoms: A structural investigation**"**, J. Vac. Sci. Technol. B 9, 2256 (1991)

[S5] Suh, J., Fu, D., Liu, X., Furdyna, J.K., Yu, K.M., Walukiewicz, W. & Wu, J.," Fermi-level stabilization in the topological insulators Bi2Se3 and Bi2Te3: Origin of the surface electron gas", Phys. Rev. B 89, 115307 (2014)

[S6] Tamtögl, A., Kraus, P. , Avidor, N. Bremholm, M ,Hedegaard, E. M. Bo, J., Iversen, B. , Bianchi, M., Hofmann, Ph., Ellis, J., Allison, W. , Benedek, G. & Ernst, W. ," Electron-Phonon Coupling and Surface Debye Temperature of Bi_2Te_3(111) from Helium Atom Scattering", Phys. Rev. B 95, 195401 (2017).

[S7] Luttinger, J.M. & Kohn, W., "Motion of Electrons and Holes in Perturbed Periodic Fields", Phys. Rev. 97, 869 (1955)

[S8] Antoncìk, E. & Landsberg P.T. ,"Overlap Integrals for Bloch Electrons Proc.Phys. Soc. 82, 337 (1963)
